# Characterization of Green and Yellow Papaya (*Carica papaya*) for Anti-Diabetic Activity in Liver and Myoblast Cells and Wound-Healing Activity in Fibroblast Cells

**DOI:** 10.3390/nu15081929

**Published:** 2023-04-17

**Authors:** Haiwen Li, Obaid Ullah Beg, Ahmed Reza Rafie, Sadia Kanwal, Alexandra Ovalle-Cisneros, Milton Omar Faison, Rafat Ali Siddiqui

**Affiliations:** 1 Food Chemistry and Nutrition Science Laboratory, Agricultural Research Station, College of Agriculture, Virginia State University, Petersburg, VA 23806, USA; hali@vsu.edu (H.L.); obaidbeg@gmail.com (O.U.B.); skanwal@vsu.edu (S.K.); 2 Cooperate Extension, College of Agriculture, Virginia State University, Petersburg, VA 23806, USA; arafie@vsu.edu; 3 Department of Biology, College of Natural Sciences, Virginia State University, Petersburg, VA 23806, USA; aova7540@students.vsu.edu (A.O.-C.); mfaison@vsu.edu (M.O.F.)

**Keywords:** glucose uptake, polyphenols, anti-oxidation, GLUT-2, triglycerides

## Abstract

Obesity and diabetes, often characterized as “metabolic syndrome”, have been recognized as two of the most important public health issues worldwide. The objective of the present research was to evaluate green and yellow papaya for anti-oxidation and anti-diabetic properties. Leaves, skin, pulp, and seed samples from papayas were freeze-dried and then extracted in water or 80% methanol. The extracts were used to determine total polyphenolic content and anti-oxidation activities, and to determine biological activities, including glucose uptake, Glut-2 expression, triglyceride reduction, and wound-healing activity. Our data demonstrated that methanol and water extracts of green and yellow papaya have similar concentrations of polyphenols in skin (10–20 mg/g dry powder), leaf (25–30 mg/g dry powder), and pulp (1–3 mg/g dry powder) fractions. However, both methanol and water extracts of seeds from yellow papaya have substantially higher concentrations of polyphenols compared to green papaya. Both water and methanol extracts of yellow papaya exhibited higher anti-oxidation activity compared to green papaya in skin (50–60%), pulp (200–300%), and seeds (10–800%). Old leaves also showed greater anti-oxidation activity (30–40%) compared to new leaves. Pulp extracts from both yellow and green papaya stimulated greater glucose uptake, but only pulp from green papaya stimulated glucose uptake in muscle cells. Similarly, pulp extract stimulated glucose transporter Glut-2 expression in liver cells. The skin, pulp, and seeds of green or yellow papaya showed triglyceride-lowering activity in liver cells by 60–80%, but samples taken from yellow papaya had a more potent effect. Seeds from both green and yellow papaya significantly stimulated the migration of fibroblasts in the wounded area by 2–2.5-fold compared to the untreated control. Consistent with these data, seeds from both green and yellow papaya also significantly stimulated collagen synthesis in fibroblast cells by almost 3-fold. In conclusion, our data indicate that different parts of papaya produce stimulatory effects on glucose uptake, Glut-2 expression, TG reduction, and wound-healing activities. This study concludes that different parts of the papaya can be beneficial for preventing diabetes and diabetes-related wound healing.

## 1. Introduction

Being overweight or obese increases the chances of type 2 diabetes. According to recent statistics, 37.3 million Americans, or 11.3% of the population, had diabetes in 2019. Its prevalence was the highest (13–16%) among African Americans and American Indians/Alaskan Natives, with more Americans diagnosed with diabetes every year (Centers for Disease Control and Prevention) [1]. Nearly 1.9 million Americans have type 1 diabetes, and about 86 million Americans aged 20 and older have prediabetes [2]. The International Diabetes Federation’s (IDF) Diabetes Atlas reported that 537 million people were diabetic in 2021 and about 6.7 million died due to diabetes-related complications [3]. One major complication of diabetes is unmanageable wounds that lead to lower-limb amputation (LLA). The reported annual incidence of LLA related to peripheral vascular disease has ranged from approximately 20 to 35 per 100,000 inhabitants [4]. Diabetes and diabetes-related complications are a serious emerging national problem in the United States, particularly in the African American population. The total cost for diabetes management was about USD 237 billion in 2017 [5]. It is important to decrease, or if possible, reverse, the occurrence of prediabetes, and to treat diabetes and its complications with less expensive and less toxic therapies. The use of dietary antioxidants and nutraceuticals is one of the strategies often pursued to reduce oxidative stress for preventing diabetes and its chronic complications [6,7].

Papaya (*Carica papaya*) is a perennial plant of tropical and subtropical regions that requires temperatures of between 21 and 33 °C and cannot tolerate temperatures less than 15 °C [8,9]. Different parts of the papaya (seeds, roots, leaves, barks, flowers, latex, and fruit) have been used in folk medicine to treat various diseases, including diabetes, cancer, and cardiovascular and infectious diseases [10]. Recent scientific studies have validated many of these traditional uses and reported anti-viral, anti-bacterial, anti-protozoal, anti-fungal, anti-inflammatory, anti-tumor, anti-hypertensive, wound-healing, neuroprotective, diuretic, abortifacient, anti-fertility, hypoglycemic, and hypolipidemic properties [11,12,13,14]. This wide range of biological activities of papaya is attributed to its content of a number of phytochemicals including flavonoids, polyphenols, alkaloids, glycosides, triterpenes, lectins, saponins, polysaccharides, vitamins, minerals, enzymes, proteins, and oils [10].

Seeds, leaves, and fruits are the most commonly used parts of the papaya for medicinal purposes. The primary components of papaya seeds are proteins, fatty acids, and phospholipids; as secondary metabolites, they also contain benzyl isothiocynate, benzyl glucosinolate, beta-sitosterol, caricin, carpaine, and enzyme myrosin [15]. The highest amount of flavonoids is contained in the papaya leaves, whereas the least amount of these secondary metabolites can be found in the skin and seeds [16]. Papaya leaf extracts have been used to treat cancer and infectious diseases [17,18]. Its vasodilating, anti-oxidation, hypoglycemic, and lipid-lowering properties are associated with lowering cardiovascular risk and treating diabetes [13,19]. The latex of papaya contains enzymes including papain, chymopapain, caricain, glycyl endopeptidase, and papaya lipase [20]. Papaya latex preparations have been used to treat tissue burns and microbial/helminthic infections [21,22]; they have also been used for insecticidal/molluscicidal activity against various pests [21,22]. The flesh from both green and ripe papaya has also been shown to have anti-hyperglycemic and anti-cancer activity [13,23,24]. The fruit also shows anti-bacterial activity against Staphylococcus aureus, Bacillus cereus, Escherichia coli, and Pseudomonas euroginosa [25,26]. In addition, the proteolytic enzymatic activity and anti-microbial activity in pulp are effective in desloughing necrotic tissue and preventing wound infection [27]. Papaya has also shown anti-hyperglycemic and hypolipidemic effects. Furthermore, studies using fermented papaya have shown that this preparation is able to reduce both basal and postprandial glycemia and improve the lipid profile. The hypoglycemic and hypolipidemic activities of papaya fruit are important yet unrecognized resources in the dietary management of diabetes, and they deserve evaluation at the cellular and molecular levels. The objective of the current study was to identify, characterize, and compare the anti-diabetic properties of green and yellow papaya.

## 2. Materials and Methods

### 2.1. Materials

Dulbecco’s Modified Eagle’s Medium (DMEM), anti-biotic/anti-mycotic solution, fetal bovine serum (FBS), and 0.25% trypsin with 0.9 mM EDTA were purchased from Invitrogen (Carlsbad, CA, USA). HepG2 and C2C12 cell lines were purchased from the American Type Culture Collection (Rockville, MD, USA), whereas Hep3B and Hu7 cells were kindly donated by Dr. Devanand Sarkar from Virginia Commonwealth University, Richmond, VA. 2-NBDG (2-(N-(7-Nitrobenz-2-oxa-1,3-diazol-4-yl)Amino)-2-Deoxyglucose) were purchased from Sigma-Aldrich (St. Louis, MO, USA). An oil red O staining kit was purchased from Lifeline Cell Technology (Frederick, MD, USA). A Triglyceride Colorimetric Assay kit was purchased from Cayman Chemical (Ann Arbor, MI, USA).

### 2.2. Papaya Samples’ Preparation

Papaya was obtained from Randolph Farm at VSU. The plants were initially grown in greenhouses and then transferred into specially designed high tunnels for growth. Green papaya and papaya leaves were harvested in October while yellow papaya was obtained at a local market. The fruits and leaves were washed with distilled water and air-dried. Green and yellow papayas were fractionated into skin, pulp, and seeds. All fractions were frozen at −80 °C and then freeze-dried in a freeze dryer (SP Scientific, Gardiner, NY, USA). The frozen samples were then ground into fine powders using a Micro Mill (Bel-Art, New Jersey, NJ) and homogenized into 20 mL of distilled water or 80% methanol via vigorous shaking for 24 h. The samples were centrifuged at 2000× *g* for 20 min to remove the undissolved material, and the soluble extract was carefully removed. The methanol extracts were dried in pre-weighed tubes using a Nitrogen Evaporator (Organomation Associates, Inc., Berlin, MA, USA), whereas the water extracts were dried in a freeze dryer (SP Scientific, Gardiner, NY, USA). The dried extract was weighed and stored at −80 °C until used.

### 2.3. Determination of Total Polyphenols

Total polyphenols (TPC) was measured using Folin and Ciocalteu’s (FC) reagent [28] with slight modification to adopt a 96-well microplate version. Total phenolic content was determined using Gallic acid as a standard curve. The analyses were performed in triplicate and data were expressed as milligrams of Gallic acid equivalents (GAE)/g of the dried sample.

### 2.4. Determination of Total Flavonoids

Briefly, Briefly, samples were nitrozalide with NaNO_2_ before adding ALCl_3_ and catechin was used as the standard as described previously [29]. Samples were read for absorption at 330. The data were expressed as μg catechin equivalent/mg dry extract.

### 2.5. Antioxidant Assays

#### 2.5.1. DPPH (2,2-Diphenyl-1-Picryhydrazyl) Assay

The DPPH assay was performed using a procedure as previously described [28]. Trolox was used to generate a calibration curve (0–70 µM). The DPPH•+ scavenging capacity of papaya extracts was demonstrated by plotting against a Trolox antioxidant standard curve. The experimen was conducted in triplicate and data were expressed as µM of the Trolox equivalents (TE)/g of the dried sample.

#### 2.5.2. FRAP (Ferric-Reducing Antioxidant Potential (FRAP) Assay

The FRAP was performed to determine the ferric-reducing power of papaya extracts as described in [30], using FeCl_3_ as the standard. Data were expressed as μM of FeCl_3_ equivalent/g dry extract.

### 2.6. Cell Culture

Human HepG2, Hep3B, and Hu7 cells were propagated in T-75 flasks using Eagle’s Minimum Essential Medium (EMEM), whereas C2C12 cells were cultured in Dulbecco’s Modified Eagle’s Medium (DMEM, 4.5 mg/mL glucose). Both media were supplemented with 1% anti-biotic/anti-mycotic solution and 10% heat-inactivated fetal bovine serum (FBS). Cells were incubated at 37 °C in a humidified atmosphere with 5% CO_2_.

### 2.7. Glucose Uptake Assays Using 2-NBDG

Glucose uptake in the presence or absence of papaya extracts was conducted using a Cayman Chemical Glucose Uptake Cell-Based Assay Kit following the manufacturer’s protocol (Ann Arbor, MI, USA). In brief, HepG2, Hep3B, Hu7 hepatic, and C2C12 cells were cultured overnight in 96-well plates at a density of 1 × 10^4^ cells per well and treated with papaya extracts for 24 h. The culture medium was then changed to glucose-free DMEM culture without papaya extracts and cultured for 6 h with 2-NBD-Glucose (200 µg/mL). At the end of the treatment, supernatant was aspirated after centrifugation. Cells were washed twice with 200 µL of cell-based assay buffer and fluorescence was detected using a plate reader (SpectraMax M5, Molecular Devices, San Jose, CA, USA) using excitation/emission of 485/650 nm.

### 2.8. Glut-2 Expression in HepG2 Cells

HepG2 cells (107/well) in 6-well plates were treated with papaya extracts (100 μg/mL) for 24 h. After treatments, cells were washed 1x and then lysed in 0.5 mL of RIPA buffer containing protease inhibitor cocktail (Roche, Indianapolis, IN, USA). Samples were used for the expression of Glut-2 using ELISA following the ThermoFisher protocol and values were normalized for protein concentration using a Pierce™ BCA Protein Assay Kit (ThermoFisher, Washington DC, USA).

### 2.9. Glut 4 Expression in C2 C12 Myoblast Cells, USA

C2C12 cells were seeded in a 6-well plate at a density of 1 × 10^4^ for 24 h. The next day, cells were treated with the papaya extract (200 μg/mL) or the vehicle control (DMSO) in 500 μL of glucose-free D, MEM for 24 h. The Glut 4 expression in C2C12 cells was determined using a quantitative real-time polymerase chain reaction (RT-PCR). Total RNA was extracted from C2C12 cells using the RNeasy mini kit (Qiagen, Germantown, MD, USA). The concentration of the RNA was determined by NanoDrop 2000 (ThermoFisher, Washington DC, USA). First-strand cDNA was synthesized from total RNA using the RT2 Easy first strand kit (Qiagen, Germantown, MD, USA). Glut-4 mRNA expression levels were analyzed via real-time PCR (QuantStudio 3, Applied Biosystems instrument, Waltham, MA, USA) under the following reaction conditions: initial denaturation at 95 °C for 5 m, followed by 40 cycles of denaturation at 95 °C for 30 s, annealing at 60 °C for 30 s, and lastly elongation at 72 °C for 30 s. The relative gene expression was quantified using the 2^−ΔΔCt^ method [31]. Glut-4 expression levels were normalized against GAPDH. The sequence of the primers employed in this study was as follows: forward primer: 5′GAGCCTGAATGCTAATGGAG3′ and reverse: 5′ GAGAGAGAGCGTCCAATGTC3′ for Glut 4; forward primer: 5′ TGAGTACGTCGTGGAGTCCA3′ and reverse primer: 5′ TAGACTCCACGACATACTCA3′ for GAPDH.

### 2.10. Triglyceride Assays

HepG2 cells were seeded and treated with papaya extracts in six-well plates as described above. The triglyceride content in the cell lysates was quantified using the Cayman Chemical triglyceride colorimetric assay following the manufacturer’s protocol (Ann Arbor, MI). In brief, cells were carefully washed twice with ice-cold PBS and harvested by collection with a rubber cell scraper. Cells were then spun down by centrifugation at 2000× *g* for 10 min at 4 °C and then re-suspended in 500 μL ice-cold diluted Standard Diluent Buffer. After sonication, cell suspension was collected via centrifugation at 2000× *g* for 10 min at 4 °C. A total of 10 μL of supernatant or positive control was added to each well and mixed with 150 μL diluted enzyme mixture containing lipoprotein lipase, glycerol kinase, glycerol phosphate oxidase, and peroxidase. Reactions were incubated for 15 min at room temperature and absorbance was measured at 550 nm using a microplate reader. Triglyceride content was expressed as mg/dL (*n* = 5).

### 2.11. Wound-Healing Scratch Assay

The experiment was performed using human fibroblast (HFF-1) cells. The cells were cultured in DMEM with 10% fetal bovine serum (FBS) in a 24-well plate and incubated in a humidified atmosphere at 37 °C and 5% CO_2_. The cells were grown to a confluent cell monolayer (24–36 h). The medium was pipetted out and discarded, and a small area was scratched using a 200 μL pipette tip. The cells were then rinsed with phosphate-buffered saline (PBS) to remove the loosened debris of the cells. Images were recorded for measuring the total wounded area. The cells were then incubated in 100 μg/mL of papaya extract and the plates were incubated at 37 °C and 5% CO_2_ in an incubator for 24 h. The scratched areas were reimaged, and the area covered by cell migration and proliferation was quantified as described previously [32].

### 2.12. Collagen Sircol Dye Binding Assay

The supernatant from the wound-healing experiments as described above was collected and centrifuged at 2500× *g* to remove debris. An aliquot of 200 μL of cell medium was used to precipitate collagen with 4 M NaCl. The collagen pellet was collected via centrifugation at 15,000× *g* for 10 min at room temperature and then the pellet was redissolved in 0.5 mL of 0.5 M acetic acid. Sircol dye reagent (Sircol dye in picric acid) was mixed with a re-dissolved collagen sample at a ratio of 1.0:0.1 mL and the solution was mixed via gently repeated inversion at room temperature for 30 min. The resulting collagen bound to Sircol was collected via centrifugation at 10,000× *g* for 10 min at room temperature. After discarding the supernatant, the pellets were redissolved in 1 mL of 1 N NaOH reagent, and the collagen concentration was determined at OD 540 against the standards [33]. An aliquot of the media supernatant was used for protein concentration determination via the Pierce BCA Protein Assays.

## 3. Results

### 3.1. Polyphenolic Contents in Green and Yellow Papaya

**Figure 1:**
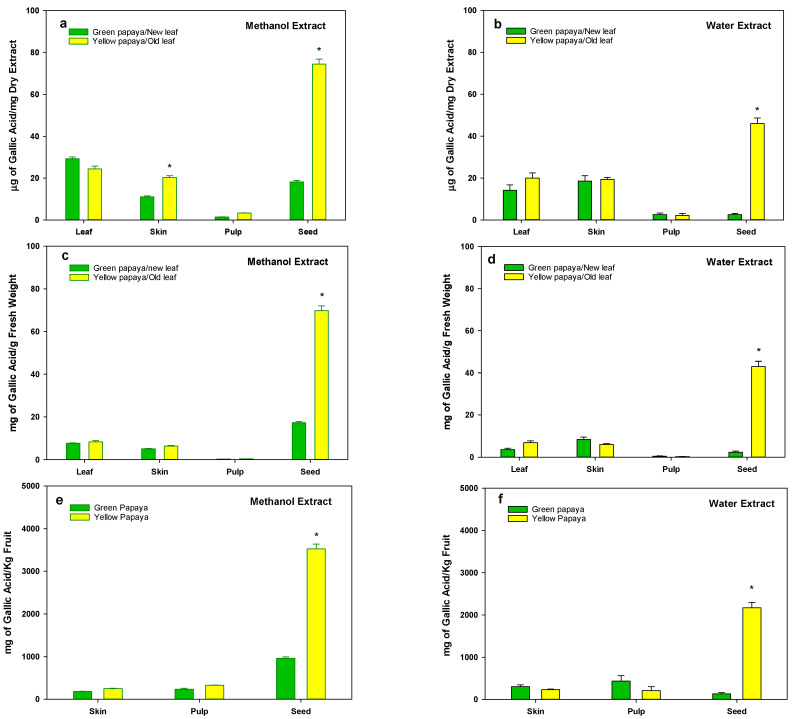
Total polyphenols in green and yellow papaya. Total polyphenols (TPC) were measured using Folin and Ciocalteu’s (FC) reagent. Total phenolic content was determined using Gallic acid as a standard. Analyses were performed in triplicate and data were expressed as milligrams of Gallic acid equivalents (GAE)/g of dried sample or fresh sample or in a 1 kg fruit. The statistical analysis was performed using Student’s *t* test between green and yellow papaya. The significant difference is represented by “*” at *p* < 0.05.

The data for the total polyphenols as shown in Figure 1a,b indicate that methanol and water extracts of green and yellow papaya have similar concentrations of polyphenols in skin (10–20 mg/g dry powder), leaf (25–30 mg/g dry powder), and pulp (1–3 mg/g dry powder) fractions. However, both methanol and water extracts of seed from yellow papaya have a substantially high concentration of polyphenols (75 or 45 mg/g dry weight in MeOH or water extracts, respectively) compared to green papaya (18 or 2.5 mg/g dry weight in MeOH or water extracts, respectively). A similar pattern was also observed when the concentration was expressed as mg/g fresh weight (Figure 1c,d). Based on the data shown in Table 1, we recalculated the amount of TPC present in different parts of papaya to be about 1 kg. Although pulp represents about 90% of papaya mass, still most of the TPC are present in the seeds of yellow papaya (Figure 1e,f); however, the methanolic seed portion contained about two times more polyphenols than that of green papaya. We also calculated the amounts of polyphenols that could be extracted from 1 kg of papaya leaves. The methanolic extracts of both old and new leaf appeared to have a higher concentration of TPC (800–900 mg/Kg leaf), whereas the water extracts of new leaves contained about 50% less TPC (375–400 mg/kg fresh leaf) compared to that of old leaves (775–800 mg/kg fresh wt).

### 3.2. Total Flavonoid Content in Green and Yellow Papaya

**Figure 2:**
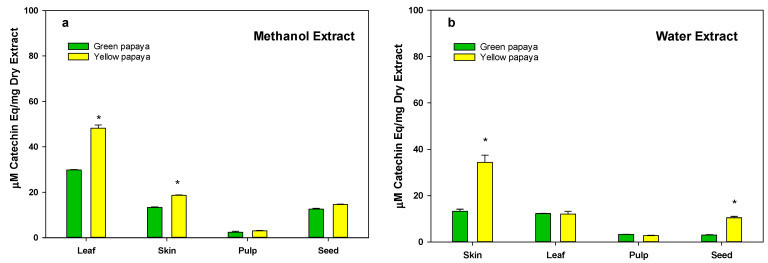
The total flavonoid content in green and yellow papaya. For the determination of total flavonoids, samples were nitrozalide with NaNO_2_ before adding ALCl_3_, and catechin was used as the standard. The data were expressed as μg catechin equivalent/mg dry extract. The statistical analysis was performed using Student’s *t* test between green and yellow papaya. The significant difference is represented by “*” at *p* < 0.05.

Data shown in Figure 2a,b indicate that, generally, leaves contain the most flavonoids in methanol extracts (30–50 μg/mg dry extract), whereas the lowest concentration was found in pulp (0.5–3 μg/mg dry extracts). Seed and skin (3–20 μg/mg dry extract) also had significantly less flavonoids than the leaf. Typically, samples of yellow papaya had more flavonoids than green papaya. 

### 3.3. Anti-Oxidation Activity in Green and Yellow Papaya Fractions

**Figure 3:**
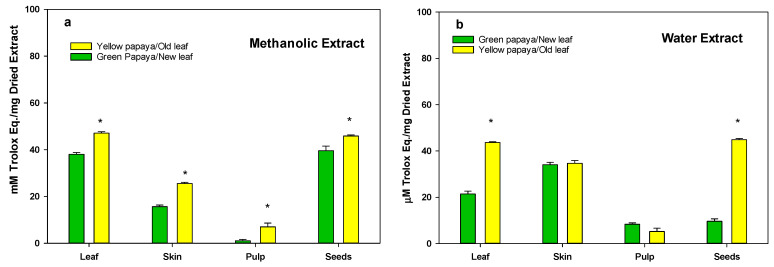

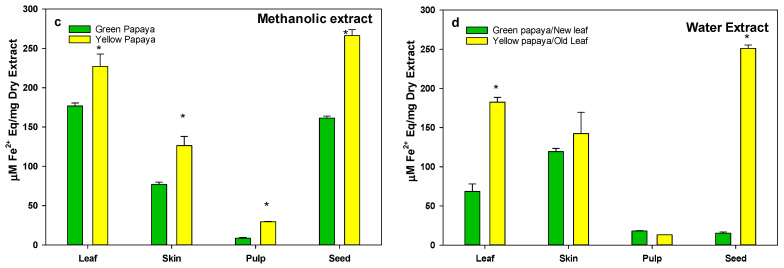
Anti-oxidation activity in green and yellow papaya fractions. The anti-oxidation activity in green and yellow papaya was determined using DPPH (2,2-diphenyl-1-picryhydrazyl) and FRAP (ferric-reducing antioxidant potential (FRAP)) assays as described in the text. The experiment was conducted in triplicate and data were expressed as µM of Trolox equivalents (TE)/g of dried sample (**a**,**b**) or μM of FeCl_3_ equivalent/g dry extract (**c**,**d**). The statistical analysis was performed using Student’s *t* test between green and yellow papaya. The significant difference is represented by “*” at *p* < 0.05.

The anti-oxidation activity as determined using the DPPH and FRAP methods is shown in Figure 3a,b and Figure 3c,d, respectively. A similar pattern of anti-oxidation activity was observed using these procedures on a different papaya sample. The data indicate that both water and methanol extracts of yellow papaya exhibited higher anti-oxidation activity compared to green papaya in skin (50–60%), pulp (200–300%), and seeds (10–800%). Old leaves were also higher (30–40%) in anti-oxidation activity compared to new leaves. The highest difference in anti-oxidation activity between parts of the green and yellow papayas was found in the water extracts of seed samples, where seeds from yellow papaya exhibited 5–10-fold higher anti-oxidation activity compared to seeds from green papaya.

### 3.4. Glucose Uptake Stimulatory Activity

**Figure 4:**
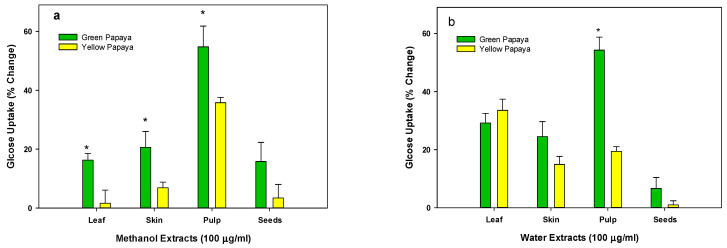
Glucose uptake by green and yellow papaya extract: The glucose uptake stimulatory activity in HepG2 liver cells was conducted using a 2-NBD-glucose, a cell-permeable glucose analog (Cayman Chemical Glucose Uptake Cell-Based Assay Kit) as described in the text. The experiment was conducted in triplicate and data were expressed as % change from vehicle-treated control cells. The statistical analysis was performed using Student’s *t* test between green and yellow papaya. The significant difference is represented by “*” at *p* < 0.05.

As shown in Figure 4a,b, the methanolic extracts of new leaf or skin, pulp, and seed from green papaya portions have almost 1.5- to 2-fold higher glucose uptake stimulatory activity in liver cells than that of old leaf or yellow papaya portions. The water extract of skin, pulp, and seed also showed a similar pattern, but a significant difference was only found with green pulp extracts where about 2.5-fold greater glucose uptake was observed compared to yellow pulp.

**Figure 5:**
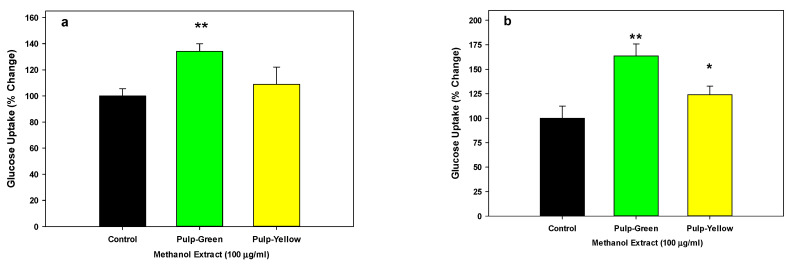
Glucose uptake by green and yellow papaya extract in Hep3B (**a**) and Hu7 (**b**) liver cells: The glucose liver cells were examined using a 2-NBD-glucose, a cell-permeable glucose analog (Cayman Chemical Glucose Uptake Cell-Based Assay Kit) as described in the text. The experiment was conducted in triplicate and data were expressed as % change from vehicle-treated control cells. The statistical analysis was performed using one-way ANOVA (analysis of variance) with Tukey’s HSD post hoc test. The significant difference is represented by “*” at *p* < 0.05 or “**” at *p* < 0.01.

We further tested the glucose stimulatory activity of green and yellow pulp in other liver cell lines. Our data, as shown in Figure 5a,b, indicated that pulp from green papaya significantly stimulated glucose uptake (*p* < 0.01) by 34% and 63% in Hep3B and Hu7 liver cells, respectively. In contrast, the pulp from yellow papaya was ineffective.

### 3.5. Glucose Transporter, Glut-2 Expression

**Figure 6:**
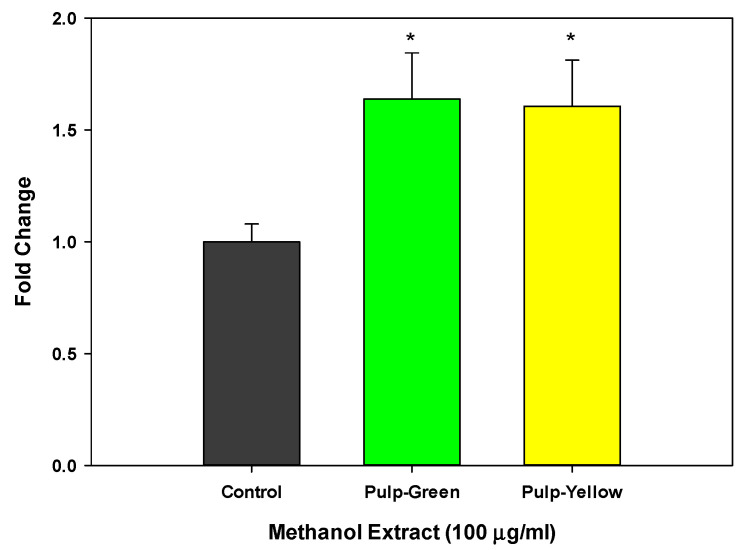
Effect of papaya pulp on Glut-2 expression. The expression of Glut-2 was determined using ELISA following steps as described in the text (ThermoFisher protocol) and values were normalized for protein concentration using a Pierce™ BCA Protein Assay Kit (ThermoFisher). The data were expressed as % change from vehicle-treated control cells. The statistical analysis was performed using Student’s *t* test between control and green or yellow papaya. The significant difference is represented by “*” at *p* < 0.05.

Since methanolic extracts of pulp fraction from green and yellow papaya exhibited a significant increase in glucose uptake, we determined the effect of papaya pulp on Glu-2 expression. The data shown in Figure 6 indicate that the methanol extracts of pulp from both green and yellow papaya similarly stimulated glucose transporter, Glut-2, expression by 75% in liver cells.

### 3.6. Glucose Uptake Activity in Myoblast Cells

**Figure 7:**
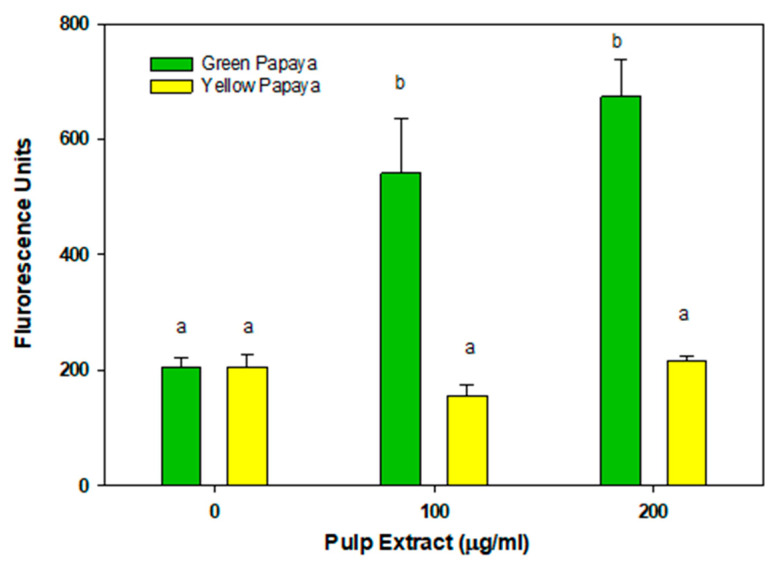
Glucose uptake by green and yellow papaya extract. The glucose uptake stimulatory activity in C2C12 myoblast cells was conducted using a 2-NBD-glucose and a cell-permeable glucose analog (Cayman Chemical Glucose Uptake Cell-Based Assay Kit) following steps as described in the text. The experiment was conducted in triplicate and data were expressed as arbitrary fluorescence units. Statistical analysis of data was performed using one-way ANOVA (analysis of variance) with Tukey’s HSD post hoc test. The statistically significant difference was within *p* < 0.05 and was represented by different letters.

**Figure 8:**
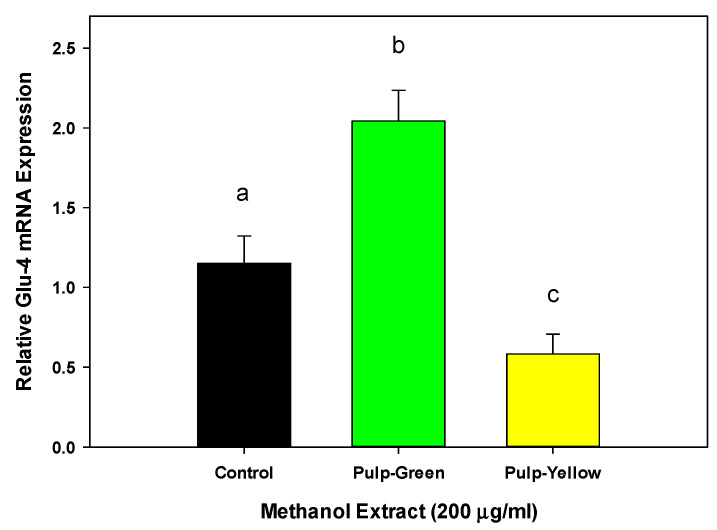
Effect of papaya pulp on Glut-4 expression. Expression of Glut-4 was determined using RT-PCR following steps as described in the text. The data were expressed as relative glut-4 mRNA expression from vehicle-treated control cells. The statistical analysis was performed using one-way ANOVA with Tukey’s HSD post hoc test (*n* = 4). The statistically significant difference at *p* < 0.05 between groups was represented by different letters.

Figure 7 shows the glucose uptake effect of the pulp extracts from green and yellow papaya in myoblasts. The pulp from the green papaya fraction stimulated glucose uptake by almost 3- to 3.5-fold in myoblast cells. In contrast to liver cells, pulp from yellow papaya did not stimulate glucose uptake in skeletal muscle cells. Consistent with glucose uptake data, green papaya extracts upregulated glut-4 expression by 2-fold (*p* < 0.05), whereas yellow papaya extracts decreased glut-4 expression by 30% (Figure 8).

### 3.7. Triglyceride Lowering Effects

**Figure 9:**
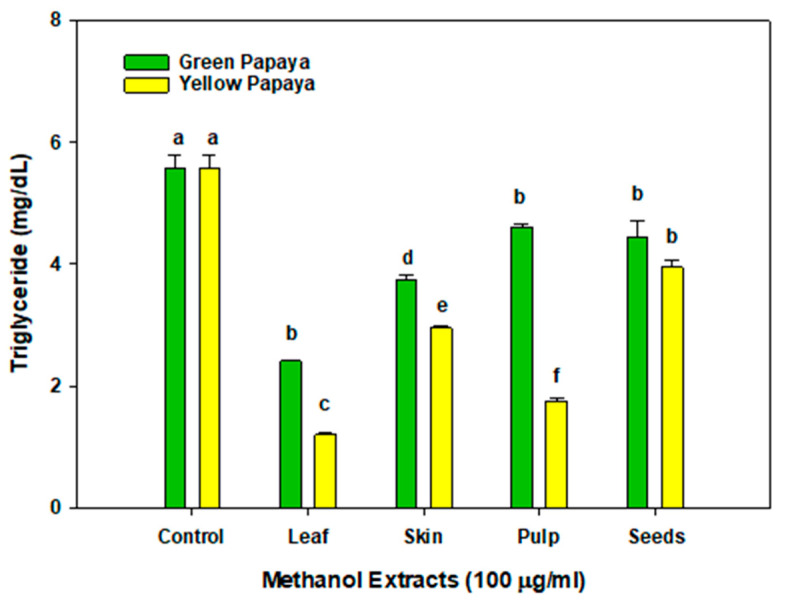
The triglyceride content in the liver HepG2 cell lysates was quantified using the Cayman Chemical triglyceride colorimetric assay following the manufacturer’s protocol (Ann Arbor, MI). Triglyceride content was expressed as mg/dL (*n* = 5). Statistical analysis of data was performed using one-way ANOVA (analysis of variance) with Tukey’s HSD post hoc test. The statistically significant difference at *p* < 0.05 within and between groups was represented by different letters compared to the vehicle-treated control.

As shown in Figure 9, new or old leaf, as well as the skin, pulp, and seeds of green or yellow papaya, showed triglyceride-lowering activity in liver cells; however, the effects of old leaf and samples from yellow papaya were more potent than those from green papaya. The most potent effect was found by old leaf and yellow pulp treatment where triglycerides in liver cells were reduced by about 60–80%. The skin of yellow papaya also reduced the triglyceride levels by almost 50%, whereas the seeds from both green and yellow papaya had a marginal effect and reduced the TG levels by 10–15%.

### 3.8. Wound-Healing Properties of Papaya Extracts

**Figure 10:**
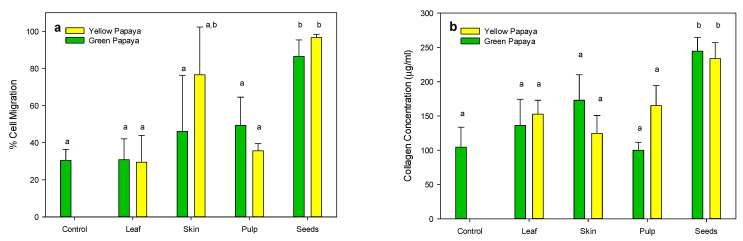
Wound-healing properties of papaya fractions. The wound-healing properties of methanolic extract of leaf, skin, pulp, and seed (100 μg/mL) were demonstrated using human fibroblast (HFF-1) cells in an in vitro scratch assay by measuring cell movement in the scratch area (**a**) and by assaying collagen synthesis (**b**) as described in the text. Statistical analysis of data was performed using one-way ANOVA (analysis of variance) with Tukey’s HSD post hoc test (*n* = 3). The statistically significant difference at *p* < 0.05 within and between groups was represented by different letters compared to the vehicle-treated control.

The data shown in Figure 10a indicate that only the seed extracts from both green and yellow papaya significantly stimulated the migration of fibroblasts in the wounded area by 2–2.5-fold compared to the untreated control. Consistent with these data, seeds from both green and yellow papaya also significantly stimulated collagen synthesis in fibroblast cells by almost 3-fold (Figure 10b).

## 4. Discussion

The present study compared the total polyphenolic compounds and anti-oxidation activities in the methanolic and water extracts of new and old leaf, as well as skin, pulp, and seed samples from green and yellow papaya. We further investigated their effects on glucose uptake, triglyceride accumulation, and wound-healing properties. As expected, the methanolic extract of yellow papaya contained higher concentrations of polyphenols, flavonoids, and anti-oxidation activity compared to green papaya; however, among all of the samples, seeds from yellow papaya had the highest TPC and anti-oxidation activity. At present, we have not yet identified the individual polyphenolic compositions. Previous studies have identified ferulic acid, caffeic acid, and rutin as being the most abundant phenolic compounds in papaya skin, whereas lycopene, β-cryptoxanthin, and β-carotene were identified as being the major carotenoids in the pulp [34]. It was also reported that the polyphenols in skin tend to decrease during ripening, whereas lycopene and β-cryptoxanthin tend to increase during ripening. The most abundant polyphenols in papaya leaf were gallic acid, caffeic acid, and rutin. In addition, catechin, naringenin, and chlorogenic and syringic acids have also been identified in leaf extracts [35]. We found that the aqueous methanol extraction resulted in a higher amount of polyphenols compared to pure water extracts. Aqueous mixtures of methanol, ethanol, ethyl acetate, and acetone have also been used by several investigators to extract polyphenols from plant materials, because the phenolic compounds have different chemical characteristics and polarities, and their solubility varies in polar and non-polar solvents [36,37].

Our data showed that methanolic samples from papaya pulp from both green and yellow papaya exhibited glucose-stimulatory activity in liver cells; however, the extract from green papaya was more effective than yellow papaya. This observation suggests that papaya pulp can be helpful in lowering blood glucose by stimulating its uptake in liver and muscle cells. Our results are consistent with another study that reported the anti-diabetic effect of ethanol extract of papaya on streptozotocin-induced diabetic mice [38]. The results of this study indicated that there was a significant decrease in the blood glucose level of the papaya-treated groups compared to the diabetic control, and papaya extract significantly increased the regeneration of the β-cells when compared to the diabetic control.

Diabetes is one of the major health conditions grouped under “metabolic syndrome”. It is an endocrine disorder, and its hallmark is accelerated blood glucose levels in the body. It is caused by a failure to either produce sufficient insulin, or to consume insulin efficiently (insulin resistance) [39]. Methods of diabetes management include lifestyle intervention and routine blood glucose monitoring to track progress toward a satisfactory reading. Pharmacological practices of diabetes are the first-line treatment, but the side effects caused by long-term medication are a severe problem. For example, thiazolidinediones (TZDs), the commonly used medicine to reduce insulin resistance, can have adverse effects such as unexpected weight increase, fluid retention, and congestive heart failure [40]. Glibenclamide (sulfonylurea family medicines) has been reported to trigger hypoglycemia, gastrointestinal symptoms, and skin problems [41,42]. Serious side effects leading to hepatitis and hepatic failure have also been reported [43,44]. An effective therapeutic approach involving medicinal plants such as papaya can be an innovating and encouraging approach for the prevention of diabetes [45].

In addition, our data showed that pulp extract from both green and yellow papaya stimulated glut-2 receptor expression in HepG2, Hep3B, and Hu7 liver cells to a similar extent. However, only pulp from green papaya upregulated glut-4 expression in myoblast cells. Glucose is the predominant energy source used by different organisms. Glucose in the bloodstream was consequentially passed through the eukaryotic cell membranes, mediated by a family of membrane glucose transporters (GLUTs). Members of the GLUT family are integral membrane proteins that are encoded by the solute carrier 2A (SLC2A) family genes [46]. There are 14 GLUT proteins in the glucose transporter family [47]. The GLUT2 gene encodes a 524 amino acid protein, and it was first identified in rat and human liver cDNA libraries [48,49]. It transports glucose at a low apparent affinity, as well as galactose, mannose, and fructose, and has a very high affinity for glucosamine [50]. GLUT2 is the major glucose transporter in the hepatocytes. The liver is the most important organ for controlling metabolic carbohydrate homeostasis, particularly carbohydrates synthesis, storage, and redistribution [51]. Improving glucose uptake into the cells is essential for glucose homeostasis rebalance and combatting type 2 diabetes [52]. Postprandial serum glucose stability was well adjusted by hepatocyte GLUTs, in which glucose was taken from the bloodstream and transformed to glycogen in the liver for energy storage. In a glucose-starving phase, the glycogen can then be converted back to glucose for the essential energy consumption [53]. “Metabolic syndrome” ailments such as diabetes destroy the balance of glucose homeostasis and cause systematic endocrine disorder, but can be corrected by the upregulation of the GLUT4 gene. A study has shown that the ethanolic extract of papaya leaf can reduce muscle insulin resistance by upregulating glut-4 in high-fat-diet- and streptozotocin-induced type 2 diabetes in rats [54]. The effects of aqueous guava leaf extract on insulin resistance and glucose transportation were evaluated in an insulin-resistant C57BL/6J mice model induced by a high-fructose–high-fat diet [55]. The results demonstrated that the oral consumption of high-dose guava leaf extract (450 mg/kg) significantly enhanced the glucose tolerance and insulin sensitivity and improved the protein levels of GLUT2 and GLUT4 in liver and skeletal muscle tissues, respectively. The anti-diabetic effects of papaya have also been revealed using an animal model of high-fructose-diet-induced type II DM, and similar results were reported by Wulansari [56]. Papaya seed extract significantly increased GLUT4 gene expression in skeletal muscle tissue from a type 2 diabetes mouse model induced by a high-fructose diet. The purpose of our current study was to decipher the anti-diabetic role of papaya in ameliorating glucose uptake and the regulation of its transportation. It was observed in our study that methanol extracts of papaya pulp showed glucose stimulatory activity in liver and muscle cells. However, the effect of green papaya was superior to that of yellow papaya. The data suggest that pulp contained compounds that potentially exert anti-diabetic effects.

Obesity is another common public health issue that is prevalent worldwide. When excessive energy consumption exceeds its expenditure, fat accumulates in the body [57,58]. Obesity causes numerous health problems, such as dyslipidemia, cardiovascular disease, cancer, and metabolic disorders, particularly insulin resistance and diabetes, amongst others [59,60]. In our present study, we examined papaya’s hypo-lipidemic effects on fat accumulation using hepatic HepG2 cells as potential anti-obesity agents. We found that leaf, skin, and pulp (except for with green papaya) significantly lowered triglyceride concentrations in liver cells. However, yellow papaya fractions were more effective than those of green papaya. Similar results were reported by Od-Ek, who evaluated the anti-obesity properties of papaya in a model on which rats were fed a high-fat diet. Their results showed that papaya reduced the upsurge of body weight, serum triglyceride, serum total cholesterol, and serum low-density lipoprotein cholesterol levels, as well as decreased serum high-density lipoprotein cholesterol levels caused by a high-fat diet [61].

The concept of “Diabesity” is associated with obesity and diabetes [62,63]. Obese individuals are prone to developing diabetes [64]. The excessive energy was stored in the white adipose tissue, but it is an important endocrine organ, not just a simple triglyceride reservoir. Adipose tissue secretes various adipokines, a protein hormone which regulates lipid metabolism and inflammatory processes [65]. A series of adipokines have been demonstrated during the past several decades, including tumor necrosis factor-α (TNF-α), leptin, Interleukin 6 (IL-6), plasminogen activator inhibitor 1 (PAI)-1, resistin, and monocyte chemoattractant protein-1 (MCP1) [66,67]. Adipokines are the trigger factors for the majority of metabolic disorders such as insulin resistance and type 2 diabetes [64,68]. The acceleration of the adipokines via adipose tissue in obese patients initiated low-grade systemic inflammation and insulin resistance, which impaired the glucose homeostasis and led to type 2 diabetes mellitus [69,70,71]. In the present study, the results showed that papaya efficiently reduced the fat accumulation and increased glucose transportation in the hepatic cells, demonstrating potential anti-obesity and anti-diabetic capacities.

On the other hand, obesity can induce higher levels of systemic oxidative stress in white adipose tissue [72]. Reactive oxygen species (ROS) molecules are extremely unstable reactive molecules and free radicals are consequentially generated by molecular oxygen [73]. ROS scrambles electrons from numerous stable molecules intracellularly [74]. They were over-produced through various biochemical pathways, including reducing the antioxidant activity and increasing chronic inflammation in adipokines, consequentially deteriorating insulin sensitivity [75,76,77]. Cellular noxiousness induced by oxygen-derived free radicals plays a critical role in the inducing of obesity and diabetes. More research has been focused on unveiling the mechanism of oxidative stress in the pathogenesis of obesity and diabetes [75]. In our current study, total phenolic content and DPPH and FRAP antioxidant assays were performed to understand the antioxidant potential of green and yellow papaya. Our results demonstrated that the seeds of yellow papaya exhibited significantly higher polyphenolic contents, which could be due to the appearance of black-brown pigment upon maturation. The methanol extract of papaya showed higher anti-oxidation activity than that of water extracts. Although, the pulp from both green and yellow papaya showed the least anti-oxidation activity; however, most of the mass of papaya lies in pulp, which potentially contributes to high total anti-oxidation activity. Papaya is a good source of antioxidant phytochemicals and contains various compounds which serve as antioxidants reducing oxidative stress [78,79,80]. Furthermore, Awodele reported that the aqueous extracts of unripe papaya pulp and leaves had a hepatoprotective effect in a Sprague Dawley rat model [81]. In their study, the serum levels of catalase (CAT), superoxide dismutase, and reduced GSH were significantly decreased. The protective effect of papaya seed extracts against oxidative stress was also reported by Salla et al. [82], showing that the reduced activities due to H_2_O_2_ treatment of superoxide dismutase (SOD), catalase (CAT), and glutathione peroxidase (GPx) were restored upon treatment in HepG2 cells.

Diabetes can damage every organ in the body. Among all potential complications stemming from diabetes, impaired wound healing is amongst the most severe [83]. A clear correlation between blood glucose and wound healing was observed. Uncontrolled high blood glucose levels cause circulation lagging and microvascular dysfunction, delaying the delivery of nutrients to wounds and postponing wound healing in diabetes patients. People with diabetes also commonly suffer from neuropathic foot ulcers. High blood glucose can damage the nerves, numb sensations in the area, and cause diabetic neuropathy [84]. Aimed at deciphering the potential use of papaya as a therapeutic alternative in promoting diabetic wound closure, a human fibroblast cell line was used for the investigation. Our results showed that the cell migration of human fibroblast cells was significantly increased when treated with papaya seed extract from both green and yellow papaya compared to the untreated control. Papaya is widely used in traditional medicine to treat various skin conditions, particularly wounds and burns, in low-income and middle-income countries. The remedial value of papaya latex on wound healing has been reported in burns on mice [85]. Hydroxyproline content was significantly increased in the papaya-treated group compared with the control group. Hydroxyproline content is an index for collagen turnover [8]. An improvement in hydroxyproline content indicates an increase in collagen synthesis. Collagen is synthesized by the healing tissue, and enhanced collagen synthesis in turn leads to boosted wound healing [86]. A similar observation was described in the Sprague Dawley rat model, where the wound-healing activity of papaya seed ethanol extract was demonstrated by a reduced wound area and increased collagen deposition [14].

## 5. Conclusions

In conclusion, green and yellow papaya are rich in polyphenols, exhibited antioxidant capacities, and may benefit free radical scavenging. Every part of the papaya fruit has distinguished biological activity. Papaya leaf, skin, and pulp exhibited strong fat-lowering effects, suggesting their potential for preventing non-alcoholic fatty liver disease and obesity. Papaya pulp showed stimulatory glucose activity in liver cells, suggesting its potential role in anti-diabetic activity. Papaya seeds stimulated cell migration in an in vitro wound assay, suggesting a potential implication in diabetes-related would healing. The present data present a preliminary investigation. Further in vivo applications of papaya extracts in high-fat diets and/or Streptozotocin-induced type II diabetes mice models, as well as in vivo wound-healing models in type II diabetic mice, are warranted to confirm our in vitro results. Our data suggest that the regular consumption of papaya can be very beneficial in preventing diabetes and obesity.

## Figures and Tables

**Table 1:** Fraction of skin, pulp, and seeds in fresh papaya.

Fresh Papaya	
Green papaya (g)	1171.48 ± 15.38
Skin (%)	3.58 ± 0.76
Pulp (%)	90.85 ± 2.12
Seeds (%)	5.57 ± 1.89
Yellow papaya (g)	1854.15 ± 24.53
Skin (%)	3.83 ± 0.50
Pulp (%)	91.12 ± 2.68
Seeds (%)	5.05 ± 2.29

## Data Availability

Data are available upon request to the first author at hali@vsu.edu.
